# Risk factors for post-traumatic stress disorder symptoms following critical illness requiring mechanical ventilation: a prospective cohort study

**DOI:** 10.1186/cc5708

**Published:** 2007-02-22

**Authors:** Timothy D Girard, Ayumi K Shintani, James C Jackson, Sharon M Gordon, Brenda T Pun, Melinda S Henderson, Robert S Dittus, Gordon R Bernard, E Wesley Ely

**Affiliations:** 1 Department of Medicine; Division of Allergy, Pulmonary, and Critical Care Medicine; Vanderbilt University School of Medicine; T-1218 MCN, Nashville, TN 37232-2650, USA; 2 Center for Health Services Research; Vanderbilt University School of Medicine; 6th Floor MCE, Suite 6100, Nashville, TN 37232-8300, USA; 3 Department of Biostatistics; Vanderbilt University School of Medicine; S-2323 MCN, Nashville, TN 37232-2158, USA; 4 Department of Psychiatry; Vanderbilt University School of Medicine; 1601 23rd Avenue South, Suite 3060, Nashville, TN, 37212, USA; 5 Veterans Affairs Tennessee Valley Geriatric Research, Education, and Clinical Center; 1310 24th Avenue South, Nashville, TN 37212-2637, USA; 6 Division of General Internal Medicine; Vanderbilt University School of Medicine; 6th Floor MCE, Suite 6000; Nashville, TN, 37232-8300, USA

## Abstract

**Introduction:**

Post-traumatic stress disorder (PTSD) has been identified in a significant portion of intensive care unit (ICU) survivors. We sought to identify factors associated with PTSD symptoms in patients following critical illness requiring mechanical ventilation.

**Methods:**

Forty-three patients who were mechanically ventilated in the medical and coronary ICUs of a university-based medical center were prospectively followed during their ICU admission for delirium with the Confusion Assessment Method for the ICU. Additionally, demographic data were obtained and severity of illness was measured with the APACHE II (Acute Physiology and Chronic Health Evaluation II) score. Six months after discharge, patients were screened for PTSD symptoms by means of the Post-Traumatic Stress Syndrome 10-Questions Inventory (PTSS-10). Multiple linear regression was used to assess the association of potential risk factors with PTSS-10 scores.

**Results:**

At follow-up, six (14%) patients had high levels of PTSD symptoms. On multivariable analysis, women had higher PTSS-10 scores than men by a margin of 7.36 points (95% confidence interval [CI] 1.62 to 13.11; *p *= 0.02). Also, high levels of PTSD symptoms were less likely to occur in older patients, with symptoms declining after age 50 (*p *= 0.04). Finally, although causation cannot be assumed, the total dose of lorazepam received during the ICU stay was associated with PTSD symptoms; for every 10-mg increase in cumulative lorazepam dose, PTSS-10 score increased by 0.39 (95% CI 0.17 to 0.61; *p *= 0.04). No significant relationship was noted between severity of illness and PTSD symptoms or duration of delirium and PTSD symptoms.

**Conclusion:**

High levels of PTSD symptoms occurred in 14% of patients six months following critical illness necessitating mechanical ventilation, and these symptoms were most likely to occur in female patients and those receiving high doses of lorazepam. High levels of PTSD symptoms were less likely to occur in older patients.

## Introduction

The life-sustaining therapies employed in the intensive care unit (ICU) commonly result in pain and anxiety as reported by survivors of critical illness [1,2]. In addition, the acute illnesses that threaten each patient's life create formidable stress. These experiences may result in long-term morbidity in survivors of critical illness, including depression, anxiety, and other psychological disorders [3]. One such psychological outcome, post-traumatic stress disorder (PTSD), has been identified in a significant portion of ICU survivors [4]. Early identification of patients who are at high risk for the development of PTSD after critical illness may facilitate the implementation of strategies focused on preventing this untoward outcome.

The current literature offers little in the way of identification of patients at high risk for PTSD after critical illness. Although female gender has long been recognized as a risk factor for the development of PTSD [5,6], the significance of gender on the development of PTSD after critical illness remains unclear. One recent study determined that ICU patients subjected to a daily interruption of sedatives developed fewer symptoms of PTSD [7]. Also, recent work has shown that ICU patients with delusional memories of their ICU stay are more likely to develop PTSD than those with factual memories [8]. Critical illness is frequently complicated by delirium [9], and delusions are a common component of delirium, suggesting that delirium may be associated with the development of PTSD. However, no previous studies of PTSD after critical illness have incorporated formal evaluations of delirium.

Therefore, this pilot investigation was conducted to identify factors associated with the development of PTSD symptoms in patients after critical illness. Specifically, we hypothesized that ICU delirium is a risk factor for the development of PTSD symptoms following critical illness and mechanical ventilation.

## Materials and methods

### Subjects

All patients who required mechanical ventilation and were admitted to the medical and coronary care ICUs of the 631-bed Vanderbilt University Medical Center (Nashville, TN, USA) between 21 February and 3 May 2001 were prospectively evaluated for enrollment. Those with neurologic disease impairing cognitive function (for example, stroke and Parkinson's disease) or mental retardation were excluded, as were non-English speakers and those with sensory deficits limiting their ability to communicate with examiners. Although no history of PTSD was identified at enrollment, it is possible that some study patients had pre-existing PTSD that was not reported; due to the non-elective nature of their ICU admissions, patients were not prospectively assessed for symptoms of PTSD prior to enrollment. The study was approved by the Vanderbilt University Institutional Review Board, and informed consent was obtained from the patients or their surrogates before study enrollment. Consent was also obtained from all patients at the six month follow-up visit. Although no outcomes data from this manuscript have been previously reported, other data from this cohort have been published [9-12].

### Procedures

Baseline data included demographics, ICU admission diagnoses, and data needed to calculate the Acute Physiology and Chronic Health Evaluation II (APACHE II) score [13] and the Charlson Comorbidity Index (calculated by the method of Deyo and colleagues [14]). While in the ICU, patients were evaluated daily for delirium with the Confusion Assessment Method for the ICU (CAM-ICU) [9,15]. The CAM-ICU had a high sensitivity (93% to 100%), specificity (89% to 100%), and inter-rater reliability (κ, 0.96; 95% confidence interval [CI] 0.92 to 0.99) when evaluated against a reference standard rater in two cohorts of medical ICU patients. Each dose of sedative (lorazepam, midazolam, and propofol) and analgesic (fentanyl and morphine) medication received was recorded daily throughout the ICU stay.

Follow-up testing was conducted six months after hospital discharge; this interval was arbitrarily defined *a priori*. Patients were screened for PTSD symptoms by means of the modified Post-Traumatic Stress Syndrome 10-Questions Inventory (PTSS-10) [16]. This two-part questionnaire assesses for memories of traumatic experiences during the ICU stay: nightmares, panic, pain, and suffocation (part A). It then measures the intensity of 10 PTSD symptoms presently experienced (that is, at or around the time of evaluation) by the patient (part B), including sleep disturbance, nightmares, depression, hyperalertness, emotional numbing, irritability, labile mood, guilt, avoidance of activities prompting recall of the traumatizing event, and muscular tension; each symptom is rated from 1 (never) to 7 (always). Total scores of more than 35 on part B predict the diagnosis of PTSD by the criteria outlined in the DSM-III (*Diagnostic and Statistical Manual of Mental Disorders, Third Edition*) [16]. The PTSS-10 has a high sensitivity (77%) and specificity (97.5%) and has been validated for use in ICU patients, with a reliability coefficient (Crohnbach's alpha) of 0.914 in this patient population [4,16]. Quality of life was assessed by means of the Short Form Health Survey-12 (SF-12) [17], and a comprehensive neuropsychological battery was performed [11]. The PTSS-10, SF-12, and the neuropsychological battery were conducted in person by a neuropsychologist (SMG) or a clinical psychologist (JCJ).

### Terminology

Although a PTSS-10 score of more than 35 predicts the diagnosis of PTSD [16], this screening instrument cannot make a formal diagnosis of PTSD. Because formal psychiatric evaluations were not carried out, results are reported in terms of PTSD symptoms rather than a diagnosis of PTSD.

### Statistical analysis

Baseline characteristics are presented using median and interquartile range for continuous variables and proportions for categorical variables. Patients evaluated at the six month follow-up and those not tested at six months were compared by means of Wilcoxon rank-sum tests for continuous variables and Fisher exact tests for categorical variables. Spearman rank correlations were employed to evaluate the correlations between PTSS-10 score and duration of delirium (defined as the total days of delirium measured in the ICU), age in years, APACHE II score, cumulative dose of sedative drug (defined as the total amount of drug received during the ICU stay separately for lorazepam, midazolam, morphine, fentanyl, and propofol), total days in the ICU, total days of mechanical ventilation, the presence of memories of traumatic ICU experiences (PTSS-10, part A), quality of life as measured by SF-12 scores, and composite neuropsychological test scores. A Wilcoxon rank-sum test was used to compare PTSS-10 scores among men and women.

To assess the independent association of each factor with PTSD symptoms, multiple linear regression was employed with PTSS-10 score as the outcome variable. Although a threshold value of 35 on the PTSS-10 has been recommended in order to maximize sensitivity and specificity, higher PTSS-10 scores across the spectrum of possible scores (10 to 70) are associated with a higher likelihood of diagnosing PTSD [16], making the PTSS-10 score a suitable continuous outcome variable. *A priori*, we chose to include age in years [18], gender [5], APACHE II score, sedative exposure [7], and days of delirium [8] in the regression model because, based on existing literature and clinical suspicion, we suspected these factors to be associated with PTSD. To assess the association between sedative exposure and PTSD symptoms, cumulative lorazepam dose was chosen based on the Spearman correlation analysis; compared with cumulative fentanyl and propofol doses, cumulative lorazepam dose was correlated most with PTSS-10 scores. Because of their possible correlation with cumulative sedative drug dose, total days in the ICU and days of mechanical ventilation were not included in the model. No variables were removed from the model. Non-linear associations between each continuous variable and PTSS-10 score were assessed by including non-linear cubic splines in the regression model. Non-linearity of the effect of age was included in the regression model because significant non-linearity was detected in its association with the outcome. To correct for possible overfitting of the regression model, penalized maximum likelihood estimation was used to allow shrinkage for non-linear effect of age. Residuals of the multiple linear regression model were examined by graphically plotting residuals against predicted values, plotting normal Q-Q plots, and using the Shapiro-Wilk test. Additionally, bootstrap model validation was used to assess the robustness of the regression model for its predictability for future data. R software version 2.11 [19], SAS version 9.0 (SAS Institute Inc., Cary, NC, USA), and SPSS version 14 (SPSS Inc., Chicago, IL, USA) were used for data analysis, and a two-sided 5% significance level was used for all statistical inferences.

## Results

Of 555 mechanically ventilated ICU patients admitted during the study period, 275 (49.5%) patients were enrolled in the study. A total of 280 patients were excluded: 86 had stroke or another primary neurologic disorder, 13 were deaf or unable to understand English, 44 died prior enrollment, 69 were extubated prior to enrollment, 27 had been previously enrolled, and consent was not obtained for 41 patients [12]. After enrollment, 96 patients died prior to hospital discharge. Of the remaining 179 patients, 23 (13%) patients died within six months of discharge, 27 (15%) were too ill to participate in follow-up evaluation or declined further participation, and 86 (48%) patients were lost to follow-up. Therefore, a total of 43 (24%) patients were evaluated six months after hospital discharge (Table 1). There were no significant differences in baseline demographics or outcome measures between the patients tested at the six-month follow-up and those not tested (for example, due to death or illness or lost to follow-up), except that hepatic and renal failure were more common in those tested (*p *= 0.003).

**Table 1:** Baseline characteristics and ICU outcomes for patients evaluated at six months and those not tested

	six-month follow-up	Not tested	
Characteristics	(*n *= 43)	(*n *= 136)	*p *value^a^
Age in years, median (IQR)	52 (39–65)	55 (42–68)	0.39
Female, percentage (number/total)	53 (23/43)	49 (66/136)	0.60
Black, percentage (number/total)	16 (7/43)	24 (33/136)	0.40
Charlson Comorbidity Index, median (IQR)	3 (2–5)	3 (1–5)	0.34
APACHE II score, median (IQR)	25 (20–31)	25 (18–31)	0.63
ICU admission diagnosis^b^, percentage (number/total)			
Sepsis and/or acute respiratory distress syndrome	42 (18/43)	49 (66/136)	0.49
Pneumonia	26 (11/43)	15 (21/136)	0.17
Myocardial infarction/Congestive heart failure	9 (4/43)	9 (12/136)	1.00
Hepatic or renal failure	12 (5/43)	1 (1/136)	0.003
COPD	2 (1/43)	10 (14/136)	0.12
Gastrointestinal bleeding	2 (1/43)	10 (14/136)	0.12
Malignancy	5 (2/43)	2 (3/136)	0.59
Drug overdose	5 (2/43)	7 (9/136)	1.00
Other	21 (9/43)	34 (46/136)	0.13
ICU length of stay in days, median (IQR)	10 (5–13)	7 (5–10)	0.08
Days on mechanical ventilation, median (IQR)	5 (3–12)	6 (3–9)	0.61
Duration of coma in days, median (IQR)	1 (0–3)	1 (0–2)	0.16
Duration of delirium in days, median (IQR)	2 (1–3)	2 (1–3)	0.39

^a^*p *values were obtained using Wilcoxon rank-sum tests for all variables except female and black/white, for which Fisher exact tests were used. ^b^Primary and secondary admission diagnoses are included, resulting in some patients being listed twice (for example, as having both sepsis and COPD). APACHE II, Acute Physiology and Chronic Health Evaluation II; COPD, chronic obstructive pulmonary disease; ICU, intensive care unit; IQR, interquartile range.

**Figure 1:**
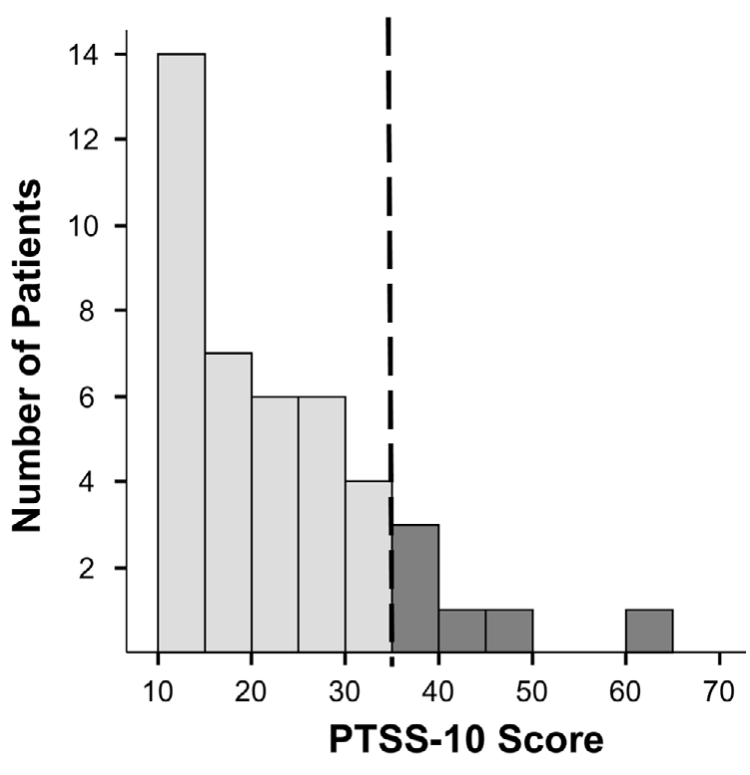
Distribution of PTSS-10 [16] scores at six-month follow-up. Median = 21; interquartile range = 14 to 30; range = 10 to 61. Vertical dashed line indicates the recommended threshold above which patients are considered to be displaying high levels of post-traumatic stress disorder symptoms. PTSS-10, Post-Traumatic Stress Syndrome 10-Questions Inventory.

At the six-month follow-up, 6 (14%) of 43 patients scored more than 35 on the PTSS-10 (Figure 1) (that is, reported high levels of symptoms consistent with PTSD). These patients reported frequent feelings of guilt (83%), mood swings (67%), and sleep disturbances (67%). Muscular tension was the symptom experienced least often (16% of patients reported frequent muscular tension). The majority of patients with PTSD symptoms at six months reported memories of panic (67%) and suffocation (50%) during the ICU stay, whereas memories of nightmares (20%) and severe pain (20%) were less common. Spearman rank correlation coefficients (rho) between PTSS-10 score and cumulative doses of sedative drugs were 0.30 for lorazepam (*p *= 0.05), -0.22 for midazolam (*p *= 0.16), 0.09 for fentanyl (*p *= 0.56), 0.07 for morphine (*p *= 0.66), and -0.16 for propofol (*p *= 0.30). Thus, cumulative lorazepam dose is included in the multivariable model.

**Figure 2:**
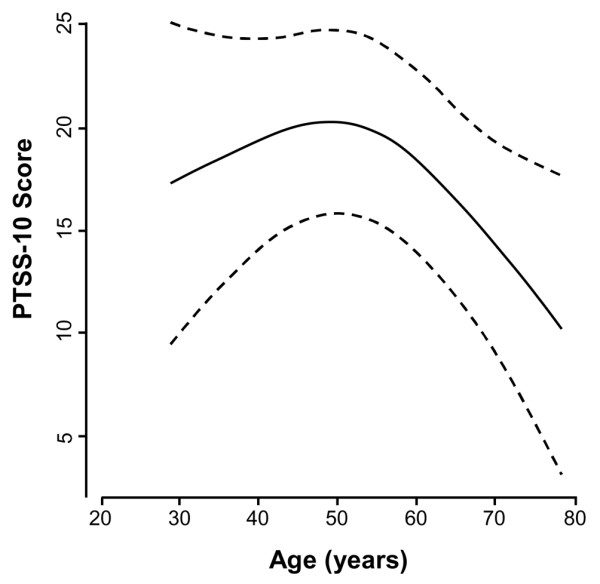
Adjusted effect of age on PTSS-10 score. The solid line indicates the predicted PTSS-10 score based on a patient's age after adjustment using multiple linear regression for APACHE II score, gender, cumulative lorazepam dose, and days of delirium. The dashed lines indicate the 95% confidence interval for the regression line. *P *= 0.04 for the effect of age and *p *= 0.04 for non-linearity, indicating PTSS-10 scores increase as age increases up to 50 years, after which PTSS-10 scores decrease as age increases. APACHE II, Acute Physiology and Chronic Health Evaluation II; PTSS-10, Post-Traumatic Stress Syndrome 10-Questions Inventory.

Results of the multivariable analysis are shown in Table 2. Women had higher PTSS-10 scores than men by a margin of 7.36 points (95% CI 1.62 to 13.11; *p *= 0.02). PTSD symptoms were less likely to occur in older patients, with symptoms declining after age 50 (*p *= 0.04) (Figure 2). The total dose of lorazepam received during the ICU stay was associated with PTSD symptoms; for every 10-mg increase in lorazepam dose, PTSS-10 score increased by 0.39 (95% CI 0.17 to 0.61; *p *= 0.04). Bootstrap validation indicated that overfitting by the regression model was minimal (2.3%), suggesting excellent robustness of prediction in future patients.

**Table 2:** Factors associated with post-traumatic stress disorder symptoms at six-month follow-up

	Univariate analysis^a^		Multivariable analysis^b^	
		
Factor	rho	*p *value	B (95% CI)	*p *value
Age in years	-0.297	0.05	Non-linear effect^c^	0.04
APACHE II	0.039	0.80	0.02 (-0.32, 0.37)	0.90
Duration of delirium in days	0.030	0.84	0.91 (-0.82, 2.63)	0.31
Total lorazepam dose (in 10 mg intervals)	0.300	0.05	0.39 (0.17, 0.61)	0.001
Female gender			7.36 (1.62, 13.11)	0.02
Median PTSS-10 score (IQR) by gender				
Female	22 (16–35)	0.06^d^		
Male	17 (12–27)			

^a^Spearman correlation coefficients (rho) unless otherwise noted. ^b^Multiple linear regression with B representing regression coefficients. ^c^See Figure 2. ^d^Wilcoxon rank-sum tests were used. APACHE II, Acute Physiology and Chronic Health Evaluation II; CI, confidence interval; IQR, interquartile range; PTSS-10, Post-Traumatic Stress Syndrome 10-Questions Inventory.

No significant correlation between PTSD symptoms and duration of delirium or APACHE II scores was demonstrated (Table 2). Additionally, PTSD symptoms were not significantly correlated with duration of mechanical ventilation (Spearman's rho, 0.034; *p *= 0.83) or with duration of ICU stay (Spearman's rho, 0.10; *p *= 0.51). Thus, the observed association between cumulative lorazepam dose and PTSD symptoms does not seem to be confounded by duration of ICU stay or mechanical ventilation. As expected, the presence of memories of traumatic ICU experiences (PTSS-10, part A) was positively correlated with PTSD symptoms (PTSS-10, part B) (Spearman's rho, 0.366; *p *= 0.02). Additionally, there was a significant inverse correlation between PTSD symptoms and quality of life as measured by SF-12 scores (Spearman's rho, -0.565; *p *< 0.0001). There was no correlation noted between PTSD symptoms and composite neuropsychological test scores (Spearman's rho, -0.079; *p *= 0.63).

## Discussion

In this investigation, high levels of PTSD symptoms after critical illness requiring mechanical ventilation were most likely to occur in female patients and in patients treated with high doses of lorazepam, whereas PTSD symptoms were less likely to occur in older patients. Understanding these risk factors may facilitate preventive strategies and direct screening for symptoms of PTSD after critical illness. In this study, 14% of patients evaluated six months after discharge reported high levels of symptoms consistent with PTSD. This coincides with the existing literature that reports a prevalence of 10% to 30% [4,7,16,18,20-25]. Despite occurring frequently, PTSD goes unrecognized in many patients. The current study confirms previous work showing that high levels of PTSD symptoms are associated with impaired quality of life [4], underscoring the importance of diagnosing and treating this disorder in survivors of critical illness.

In this study, women were significantly more likely than men to have high levels of PTSD symptoms after critical illness. The association between PTSD and female gender has been reported previously [5], but few studies have evaluated the significance of gender on the development of PTSD after critical illness. Several studies have demonstrated that women are more vulnerable to PTSD, even after controlling for differences in the type of trauma [5,6], and a higher incidence of pre-existing anxiety and/or depression disorders is postulated to play some role in the difference in PTSD rates between the sexes [6].

This study reveals a significant relationship between age and PTSD symptoms, with older patients being less likely to experience high levels of PTSD symptoms after critical illness. A non-linear relationship between age and PTSD symptoms was observed, but caution is appropriate in interpreting this finding because of the small number of younger patients studied and the results of previous research. For example, Scragg and colleagues [18] evaluated 80 ICU patients for symptoms of PTSD and reported that scores on the screening instrument were inversely correlated with age (*p *= 0.05). Rattray and colleagues [23] similarly found that symptoms of anxiety (*p *= 0.04) and avoidance (*p *= 0.01) were inversely correlated with age 12 months after discharge in 80 ICU survivors. In the current study, older patients were significantly less likely than middle-aged patients to have high levels of PTSD symptoms. Several possible explanations for this relationship exist. Although each patient studied was mechanically ventilated, older patients are less likely to receive aggressive interventions that may predispose them to the development of PTSD [26]. Additionally, given that older patients may have multiple comorbidities and a history of hospitalization, they may be less likely to view critical illness as a traumatic event.

We hypothesized that patients who experienced longer periods of delirium would be more likely to develop high levels of PTSD symptoms after critical illness, but the data do not support this hypothesis. Jones and colleagues [8] have demonstrated that the recall of delusions rather than factual memories of the ICU experience is associated with the development of PTSD symptoms. Their study assessed 45 patients after ICU discharge and revealed that patients with delusional memories and no recall of factual events in the ICU were more likely to develop PTSD symptoms than those patients with factual memories (*p *< 0.0001). These data [8] suggest that periods of delirium, with associated delusions, may predispose patients to PTSD whereas periods of alertness, which allow for the consolidation of factual memories, may protect patients from developing PTSD-related symptoms after discharge. However, the association between days of delirium and PTSD symptoms in this study was not statistically significant (*p *= 0.31).

Cumulative lorazepam dose correlated with PTSD symptoms. Although Nelson and colleagues [27] studied 24 survivors of acute respiratory distress syndrome and noted that days of sedation correlated with symptoms of both PTSD (*p *= 0.006) and depression (*p *= 0.007), the current investigation is the first to report an association between sedative dose and PTSD symptoms. However, it cannot be concluded from these analyses that lorazepam causes PTSD. The possibility exists that ICU patients who demonstrate symptoms of anxiety during their ICU stay are likely to receive higher sedative doses than those patients who are not anxious. Therefore, high lorazepam doses may identify those ICU patients with acute stress disorder, a known risk factor for PTSD [28].

Although the administration of lorazepam may lead to more PTSD symptoms or alternatively may identify anxious ICU patients, the daily interruption of sedatives may facilitate periods of alertness and reduce the risk of PTSD. Kress and colleagues [7] evaluated 32 patients who were randomly assigned to the daily interruption of sedatives or standard sedation to determine the long-term psychological effects of this intervention. The 13 patients who had been treated with daily interruption of sedation had better Impact of Events scores (11.2 versus 27.3, *p *= 0.02) and a lower incidence of PTSD (0% versus 32%, *p *= 0.06). Further study of the effect of the daily interruption of sedation on the development of PTSD is needed.

Limitations of the current study warrant comment. Because the PTSS-10 does not make a formal diagnosis of PTSD, the results of this study may not be generalizable to the clinical syndrome of PTSD. Also, the PTSS-10 does not assess for delusional memories. Data on the frequency of delusional memories, such as those provided by the ICU Memory tool [29], would have allowed for more in-depth analysis regarding the relationship between delusional memories, delirium, and PTSD symptoms. There were a significant number of patients lost to follow-up. Analysis suggests that baseline and outcome characteristics were similar between those patients lost to follow-up and those evaluated at six months (Table 1), but this does not rule out the possibility of selection bias. In fact, 'avoidance of activities prompting recall of traumatizing events' is a symptom of PTSD, and patients experiencing this symptom may have been less likely to return for follow-up testing. Thus, this study may underestimate the prevalence of PTSD after critical illness. Also, the findings regarding risk factors might have differed if all survivors had been evaluated. It was not systematically determined whether any patient sought psychiatric care prior to the six-month follow-up, and follow-up was limited to a single visit. Therefore, it is possible that some patients experienced PTSD symptoms prior to follow-up but that psychiatric treatment resulted in the resolution of such symptoms prior to testing at six months. Also, no evidence exists to define the ideal follow-up interval after which to screen for PTSD symptoms. Therefore, it is possible that screening after a shorter interval would have identified a higher number of patients with PTSD symptoms. Because of the non-elective nature of critical illness, it could not be prospectively confirmed that patients did not have PTSD prior to ICU admission. This diagnosis was not reported by family members and was not recorded in the medical record for the patients in this study. Finally, no data were collected regarding corticosteroid and beta-blocker administration, two possible confounders [30,31]. This planned pilot investigation was limited by a small sample size, and a larger study to confirm these findings is warranted.

## Conclusion

This study shows that high levels of PTSD symptoms occurred in one out of every seven patients six months following critical illness and mechanical ventilation. High levels of PTSD symptoms were most likely to occur in females and less likely to occur in older patients. Additionally, lorazepam dose in the ICU was associated with PTSD symptoms at follow-up, although causation cannot be assumed. A significant minority of patients who survive critical illness will develop symptoms of PTSD; screening for these symptoms and warning all patients about the possibility of experiencing such symptoms is prudent. Knowledge of the risk factors demonstrated in this study may facilitate identification of PTSD after critical illness. However, it is unclear what component (or components) of ICU experience (for example, the critical illness itself or treatments rendered) may contribute to the development of PTSD. The current data cannot help to answer this question, and this is an important area to be addressed by future studies. Also, additional studies are needed before firm conclusions can be made regarding the relationship between ICU delirium and the development of PTSD after critical illness.

## Key messages

• High levels of PTSD symptoms occur in nearly 20% of patients after critical illness requiring mechanical ventilation.

• High levels of symptoms of PTSD in this population are more likely among females and those treated with high doses of lorazepam.

• Older patients are less likely to have high levels of PTSD symptoms after critical illness.

## Abbreviations

APACHE II = Acute Physiology and Chronic Health Evaluation II; CAM-ICU = Confusion Assessment Method for the Intensive Care Unit; CI = confidence interval; ICU = intensive care unit; PTSD = post-traumatic stress disorder; PTSS-10 = Post-Traumatic Stress Syndrome 10-Questions Inventory; SF-12 = Short Form Health Survey-12.

## Competing interests

The authors declare that they have no competing interests.

## Authors' contributions

EWE, JCJ, SMG, and BTP participated in study conception and design, collected the data, and participated in interpretation of the results and in critical revision of the manuscript. AKS analyzed the data and participated in interpretation of the results and in critical revision of the manuscript. TDG analyzed the data, participated in interpretation of the results, drafted the manuscript, and participated in critical revision of the manuscript. MSH, RSD, and GRB participated in interpretation of the results and in critical revision of the manuscript. All authors read and approved the final manuscript.
